# Supplementary material for: Splice-Junction-Based Mapping of Alternative Isoforms in the Human Proteome
Source: Cell Rep. Author manuscript; Available in PMC 2020 Jan 15. (PMC6961840; doi:10.1016/j.celrep.2019.11.026)
Supplement: 2 [file NIHMS1546469-supplement-2.zip › DF1/PXD009737/TestisTrypsin/TestisTrypsin_61_NEK11_EPTLQNHGYFIETCVSGSTAISTYPK.pdf]

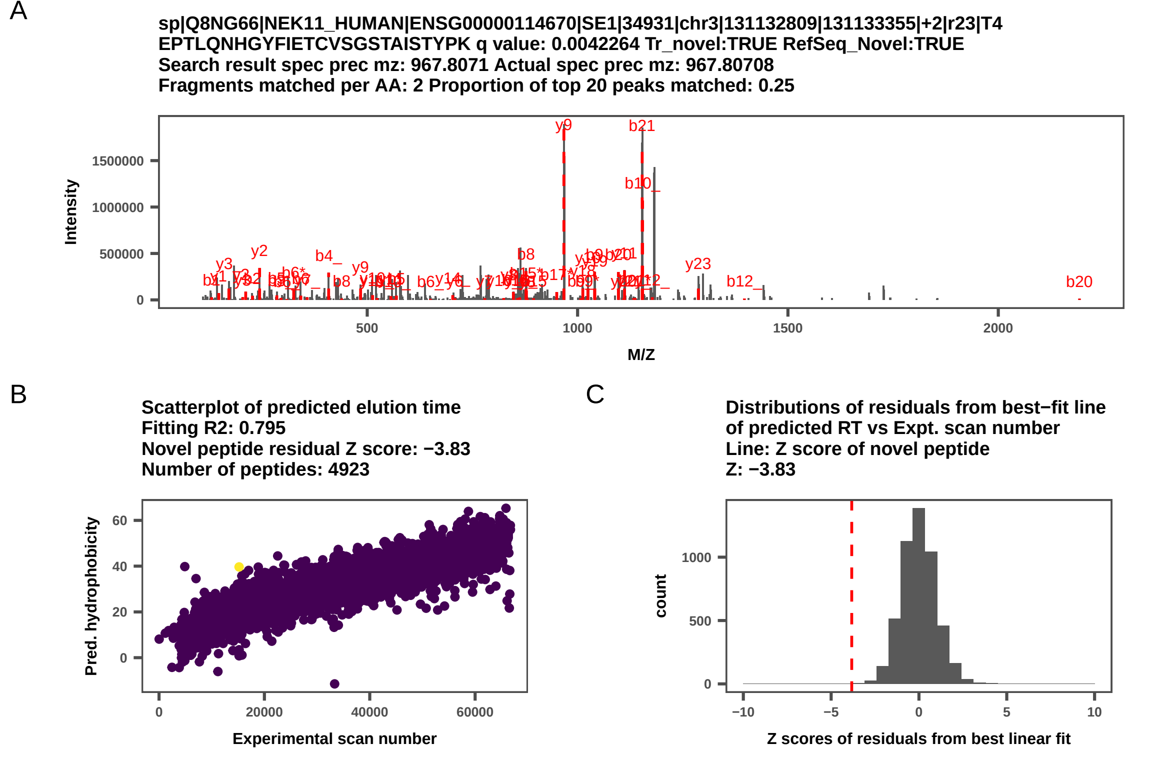
A
sp|Q8NG66|NEK11_HUMAN|ENSG00000114670|SE1|34931|chr3|131132809|131133355|+2|r23|T4
EPTLQNHGYFIETCVSGSTAISTYPK q value: 0.0042264 Tr_novel:TRUE RefSeq_Novel:TRUE
Search result spec prec mz: 967.8071 Actual spec prec mz: 967.80708
Fragments matched per AA: 2 Proportion of top 20 peaks matched: 0.25
Intensity
1500000
1000000
500000
0
M/Z
500
1000
1500
2000
y9
b21
b10_
y2
y3
b4_
b8
y23
b12_
b20
B
Scatterplot of predicted elution time
Fitting R2: 0.795
Novel peptide residual Z score: −3.83
Number of peptides: 4923
Pred. hydrophobicity
60
40
20
0
Experimental scan number
0
20000
40000
60000
C
Distributions of residuals from best−fit line
of predicted RT vs Expt. scan number
Line: Z score of novel peptide
Z: −3.83
count
1000
500
0
Z scores of residuals from best linear fit
−10
−5
0
5
10
